# Dynamic Innate Immune Responses of Human Bronchial Epithelial Cells to Severe Acute Respiratory Syndrome-Associated Coronavirus Infection

**DOI:** 10.1371/journal.pone.0008729

**Published:** 2010-01-15

**Authors:** Tomoki Yoshikawa, Terence E. Hill, Naoko Yoshikawa, Vsevolod L. Popov, Cristi L. Galindo, Harold R. Garner, C. J. Peters, Chien-Te (Kent) Tseng

**Affiliations:** 1 Department of Microbiology and Immunology, University of Texas Medical Branch, Galveston, Texas, United States of America; 2 Department of Pathology, University of Texas Medical Branch, Galveston, Texas, United States of America; 3 Center for Biodefense and Emerging Infectious Diseases, University of Texas Medical Branch, Galveston, Texas, United States of America; 4 The McDermott Center for Human Growth and Development, University of Texas Southwestern Medical Center, Dallas, Texas, United States of America; University of Giessen Lung Center, Germany

## Abstract

Human lung epithelial cells are likely among the first targets to encounter invading severe acute respiratory syndrome-associated coronavirus (SARS-CoV). Not only can these cells support the growth of SARS-CoV infection, but they are also capable of secreting inflammatory cytokines to initiate and, eventually, aggravate host innate inflammatory responses, causing detrimental immune-mediated pathology within the lungs. Thus, a comprehensive evaluation of the complex epithelial signaling to SARS-CoV is crucial for paving the way to better understand SARS pathogenesis. Based on microarray-based functional genomics, we report here the global gene response of 2B4 cells, a cloned bronchial epithelial cell line derived from Calu-3 cells. Specifically, we found a temporal and spatial activation of nuclear factor (NF)κB, activator protein (AP)-1, and interferon regulatory factor (IRF)-3/7 in infected 2B4 cells at 12-, 24-, and 48-hrs post infection (p.i.), resulting in the activation of many antiviral genes, including interferon (IFN)-β, -λs, inflammatory mediators, and many IFN-stimulated genes (ISGs). We also showed, for the first time, that IFN-β and IFN-λs were capable of exerting previously unrecognized, non-redundant, and complementary abilities to limit SARS-CoV replication, even though their expression could not be detected in infected 2B4 bronchial epithelial cells until 48 hrs p.i. Collectively, our results highlight the mechanics of the sequential events of antiviral signaling pathway/s triggered by SARS-CoV in bronchial epithelial cells and identify novel cellular targets for future studies, aiming at advancing strategies against SARS.

## Introduction

Severe acute respiratory syndrome (SARS), caused by a novel human coronavirus (CoV), has established itself as a fatal human respiratory disease [1], [2], [3], [4]. SARS-CoV is transmitted through virus-laden droplets, and likely also via either the aerosol or fecal-oral routes, with the lungs as its main pathological target. While the exact mechanism of SARS pathogenesis remains unknown, pathological examination of lung biopsies and autopsy specimens from SARS patients revealed “diffuse alveolar damage” of varying stages and severities, with extensive disruption of epithelial cells and accumulation of reactive macrophages (MΦs), accompanied by the presence of hemophagocytic syndrome in patients who succumbed to the disease [5], [6], [7], [8]. Strikingly, pulmonary manifestations of SARS patients usually occurred after the clearance of viremia and often in the absence of other opportunistic infections. Taken together, these observations have led to the hypothesis that SARS pathogenesis might stem from ill-regulated and often excessive inflammatory responses within the lungs [5]. The likelihood of SARS being an immune-mediated disease was further supported by reports, within the circulation and the lungs of patients affected by SARS, of highly elevated expressions of various inflammatory mediators, including interleukin (IL)-1, -6, -8; CXCL-10/Interferon-inducible Protein (IP)-10; CCL2/Monocyte Chemoattractant Protein (MCP)-1; CCL5/Regulated on Activation, Normal T Expressed and Secreted (RANTES); and CXCL9/Monokine Induced by interferon-Gamma (MIG) [9], [10], [11], [12], [13], [14]. Such an exacerbated cytokine response was subsequently demonstrated in experimentally infected mice, especially those transgenically expressing human angiotensin-converting enzyme 2 (hACE2) viral receptor [15], [16], [17].

In contrast to the prominently elevated cytokine response, it has been rather challenging to detect any significant response of type I IFNs in individuals and mice infected by SARS-CoV [14], [15], [16]. Such a failure of SARS-CoV in inducing readily detectable type I IFN responses was subsequently demonstrated in many *in vitro* studies by using various cell types of non-pulmonary origins, including African green monkey kidney cells (Vero cells), human peripheral blood mononuclear cells (PBMC), intestinal epithelial Caco-2 cells, hepatoma Huh7 cells, and embryonic kidney (HEK) 293 cells [14], [18], [19], [20], [21]. Because type I IFNs have been shown to be effective against SARS-CoV infection, both *in vitro* and *in vivo* [22], [23], [24], [25], [26], the deficient response of type I IFNs in infected hosts has led to the hypothesis that SARS-CoV has evolved strategies to evade this potent IFN-related innate antiviral response. Indeed, it was subsequently demonstrated that SARS-CoV-encoded ORF3b, ORF6, ORF7, nucleocapsid (N), nsp1, and, most recently, that membrane (M) [27] proteins could function as antagonists of the host antiviral defenses by interrupting the IRF-3-STAT axis of the IFN-related antiviral pathway, promoting degradation of cellular RNAs, and inhibiting IFN production by interfering with the formation of TRAF3.TANK.TBK1/IKKepsilon (ε) complex, respectively [28], [29], [30], [31], [32]. Furthermore, it has been suggested that SARS-CoV and other members of the Group 2 CoVs, such as mouse hepatitis virus (MHV), could effectively evade IFN-related antiviral responses by actively avoiding the recognition of their replicative RNAs by the host innate sensing mechanism/s [33], [34].

Human airway epithelium is likely one of the initial sites of SARS-CoV infection [35]. In addition to functioning as physical and mechanical barriers that separate and eliminate inhaled harmful infectious and non-infectious materials, lung epithelial cells can directly respond to respiratory infections by secreting various molecules that serve to initiate, amplify, and/or sustain host inflammatory responses [36], [37]. In this regard, we have recently shown that IL-6 and/or IL-8 released by SARS-CoV-infected human bronchoepithelial Calu-3 cells could exacerbate host innate inflammatory responses, in part, by modulating the intrinsic functions of macrophages (MΦ) and dendritic cells (DC) [38]. Thus, a thorough understanding of how human lung epithelial cells respond to SARS-CoV infection is crucial, not only for advancing our knowledge of SARS pathogenesis, but also for identifying novel and highly valuable cellular targets for innovative interventions against SARS.

The scarcity of and, most importantly, the highly heterogeneous nature of normal human bronchial epithelial (NHBE) cells with regard to their ACE2 expression and, thus, permissiveness to SARS-CoV infection [39], [40] (Tseng, C.K. et al., unpublished observation) greatly limits their use in exploring the genome-wide responses to viral infections. Although human bronchial epithelial Calu-3 cells, like NHBE cells, are heterogeneous, their availability and well-characterized interaction with SARS-CoV [41] make them a better choice than NHBE cells to underscore the innate antiviral signaling pathway/s induced by SARS-CoV in pathologically relevant cells. However, the fact that only up to 30% of Calu-3 cells expressed ACE2 at different intensities [41] compromises their usefulness in underscoring host innate antiviral signaling pathway(s) explicitly triggered by SARS-CoV. To overcome this, we established clonal derivatives of Calu-3 cells by standard limiting dilution. Based on their intense, homogeneous, and, most importantly, stable expression of ACE2 over various passages, we chose the 2B4 clone to explore the innate epithelial signaling pathway/s elicited by SARS-CoV by microarray-based functional genomics. Here, we have analyzed the global gene responses of 2B4 cells over time in response to SARS-CoV infection. In an attempt to clarify the characteristics of the innate antiviral responses triggered by SARS-CoV, we also simultaneously analyzed the gene expression profile of 2B4 cells infected by Dhori virus (DHOV), an orthomyxovirus known to productively infect 2B4 cells, leading to profound production of IFNs and other inflammatory cytokines (Hill, et al., manuscript in preparation). Contrary to the prevailing *in vitro* studies emphasizing the lack of readily detectable IFN responses, we find that 2B4 cells can mount an active response. However IRF-3/7-mediated IFN-β and, especially, IFN-λ responses were delayed, relative to those of NFκB- and/or AP-1 (ATF2/c-Jun)-mediated proinflammatory responses, at both the transcriptional and translational levels. The ability of 2B4 cells to activate IFN-related signaling pathway/s in response to SARS-CoV infection is further verified by the subsequent expression of many interferon-stimulated genes (ISGs). While SARS-CoV infection was capable of activating the aforementioned genes related to innate antiviral responses, the magnitude of their expression was noticeably not as high as those induced by DHOV. We also noticed that the translational machinery for IFN-β and IFN-λ2 mRNA transcripts in 2B4 cells was not as efficient as those of IFN-λ1, IL-6, IL-8, and, especially, CXCL-10/IP-10. Interestingly, such a SARS-CoV-associated inefficiency in translating genes encoding for IFN-β, IFN-λ1, and IFN-λ2 was not observed in DHOV-infected 2B4 cells. Finally, we found, for the first time, that IFN-λ1 and/or -λ2 (Type III IFNs) exert a protective role, either alone or in combination with an otherwise ineffective IFN-β, against SARS-CoV in a dose-dependent manner. Taken together, these data offer compelling evidence that human bronchial epithelial cells are capable of promoting active, but delayed, IFN-related antiviral responses, thus providing new insight into SARS pathogenesis.

## Results

### Establishment and Characterization of 2B4 Cells and Their Susceptibility to SARS-CoV Infection

**Figure 1:**
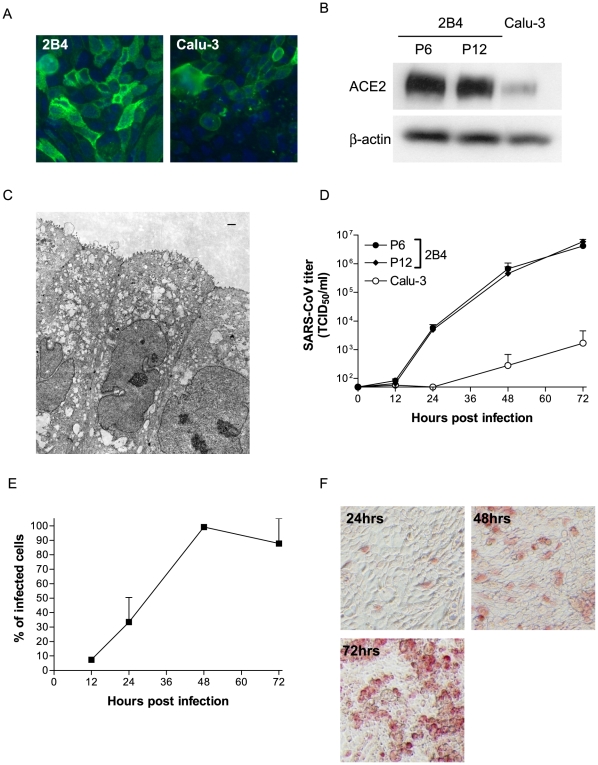
Characteristics of 2B4 cells clonally derived from human bronchial epithelial Calu-3 cells. Expressions of the viral ACE2 receptor in indicated passages of 2B4 cells and their parental Calu-3 cells were assessed by standard IHC (A) and Western blot analysis (B), whereas the morphological features of polarized 2B4 cells were assessed by TEM (C), as described in Materials and Methods. The images were taken at 6,270 magnifications. The scale bar represents 1 µm. To compare the permissiveness of 2B4 cells to their parental Calu-3 cells, confluent 2B4 cells, at passages #6 and #12, and Calu-3 cells were subjected to SARS-CoV (MOI = 0.1). The growth kinetics of SARS-CoV in culture supernatant and proportion of SARS-CoV-infected 2B4 cells were assessed at indicated time points by the standard Vero E6-based infectivity assay of the resulting cell-free supernatants (D) and infectious center assay (E). Finally, 2B4 cells (passage #6) were infected with SARS-CoV (MOI = 0.1) for 24, 48, and 72 hrs before being fixed with 4% paraformaldehyde for monitoring the morphological changes of infected cells, as visualized by the expression of SARS-CoV NP protein (red) by using the standard IHC (F).

We employed the standard limiting dilution technique to establish clonal derivatives of Calu-3 cells, as described in Materials and Methods. Based on their ACE2 expression and permissiveness to productive SARS-CoV infection, 18 out of a total of 26 clones established exhibited an array of varying intensities of ACE2 expression and permissiveness to SARS-CoV, ranging from intermediate-to-low levels, whereas the remaining eight clones revealed increased ACE2 expression and susceptibility to SARS-CoV infection, when compared to their parental Calu-3 cells (data not shown). Among those clones that were highly permissive to SARS-CoV, we chose cells of the 2B4 clone for a detailed characterization with regard to the stability of ACE2 expression over different passages and the susceptibility to productive SARS-CoV infection. As shown in Figure 1A, results of IHC staining revealed that the ACE2 expression of 2B4 cells (passage #6) was much more intense than that of the parental Calu-3 cells. Such an enhanced ACE2 expression of 2B4 cells, relative to Calu-3 cells, was confirmed by Western blot analysis (Figure 1B). Importantly, the trend of the intense ACE2 expression of 2B4 cells appeared to be stable, as cells derived from two different passages (i.e., #6 and #12) exhibited little difference, if any, in the expression of ACE2 protein. The parental Calu-3 cells, wich originated from a human pulmonary adenocarcinoma, have been well characterized as non-ciliated human bronchial epithelial cells with the expression of many markers of serous gland cells and the formation of tight junction (TJ) complexes [41], [42], [43], [44]. The morphology of 2B4 cells grown in the membrane inserts was subsequently examined by TEM. As shown in Figure 1C, 2B4 cells, like parental Calu-3 cells, appeared to have a morphology resembling that of non-ciliated pseudostratified columnar epithelial cells with the expression of microvilli on the apical surface and the formation of TJ complexes.

The permissiveness to SARS-CoV infection of 2B4 cells (passages #6 and #12) and Calu-3 cells was investigated over time, and the results depicted in Figure 1D. It became clear that infected 2B4 cells (MOI = 0.1) of either passage were equally capable of promoting a strikingly more intense production of progeny viruses than their parental Calu-3 cells. The kinetics of viral replication in 2B4 cells (passage #8) were also evaluated by the infectious center assay, as well as the standard IHC to estimate the percentage (%) of infected cells and examine the morphological changes of infected cells, respectively. As shown in Figure 1E, infected cells gradually increased from 12 hrs (i.e., ∼8%) to 24 hrs (i.e., ∼30%), reaching 100% at 48 hrs. Similarly, the expression of the SARS-CoV N protein, as revealed by the IHC, was readily detectable in infected 2B4 cells at 24 hrs, significantly increased at 48 hrs, and reached the maximum at 72-hrs p.i., accompanied by the appearance of rounded and enlarged cells (CPE) (Figure 1F), some of which became detached from the culture vessel (data not shown). Taken together, these results indicated that 2B4 cells are homogeneous with regard to their stability of ACE2 expression and permissiveness to SARS-CoV infection, thereby providing a sensitive, pathologically relevant *in vitro* model for characterizing the host innate antiviral signaling pathway/s explicitly triggered by SARS-CoV.

### Global Gene Expression of 2B4 Cells in Response to SARS-CoV Infection

We employed a cDNA microarray to analyze the patterns of the global gene expression of 2B4 cells in response to SARS-CoV, as the first step to explore the likely antiviral signaling pathway/s. To ascribe unique properties of SARS-CoV-induced innate responses (if any), it would be ideal to compare to those elicited by another strain of human coronavirus (HCoV), e.g., 229E and OC43. Unfortunately, neither HCoV-229E nor -OC43 could productively infect Calu-3 cells (data not shown), making such a comparison unlikely. Because DHOV, a proposed orthomyxoviral surrogate of the highly pathogenic avian influenza H5N1 virus [45], [46], productively infected 2B4 cells, resulting in highly intense secretion of Type I IFN and other innate inflammatory mediators (Hill et al., unpublished data), we compared the global gene expression of 2B4 cells in response to SARS-CoV versus DHOV. While the microarray-based analysis of the temporal gene expression of 2B4 cells in response to SARS-CoV or DHOV infection has been performed simultaneously within the same experimental setting, most of the results relevant to DHOV infection, unless indicated otherwise, are the subject of a separate manuscript (Hill et al. in preparation). More specifically, we were particularly interested in comparing virally induced genes that are either encoding for or relevant to the expression of IFN and other inflammatory cytokines as SARS pathogenesis has been proposed to stem from the combination of barely detectable, if any, IFN and exacerbated cytokine responses in patients severely affected by SARS-CoV infection [14], [47].

**Figure 2:**
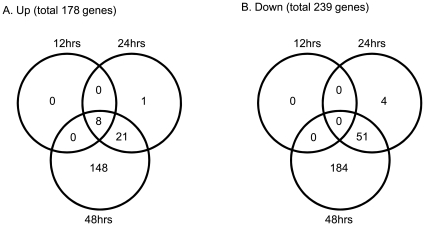
Temporal and overlapping gene expression of 2B4 cells triggered by SARS-CoV infection. Confluent 2B4 cells grown in T-75 flasks were infected with SARS-CoV at MOI = 0.1 or remained uninfected (as controls). The cells were lysed for extracting total RNAs for the subsequent microarray analysis by using Affymetrix Genechips. After the stringent pairwise comparisons and the statistical analysis, genes whose expressions were significantly altered (e.g., fold-change ≥1.5 and at least 50% greater in magnitude than any change observed between control samples, *p*<0.05) in SARS-CoV-infected versus uninfected 2B4 cells were selected for the additional analyses, as described in Materials and Methods. Two Venn diagrams were created to reflect the temporal and overlapping expressions of those up-regulated (A) and down-regulated genes (B), respectively.

The temporal expression of host genes was determined by comparing the relative abundance of specific mRNA in mock versus SARS-CoV-infected versus DHOV-infected 2B4 cells (MOI = 0.1), harvested at 12, 24, and 48 hrs p.i. The microarray-based study of global gene responses was performed in triplicate for each time point, yielding 27 arrays for analysis. Only those genes whose expressions were significantly modulated (i.e., 1.5-fold and p<0.05, when compared to those of mock-infected controls) in all of the replicates (N = 3) analyzed at each time point were selected for further investigation. The kinetics of viral replication was also determined at each time point to correlate the levels of viral infection to the extent of host gene responses. We constructed a Venn diagram, based on the results of the stringent microarray analysis, to roughly reflect the temporal changes in the gene expression of SARS-CoV-infected 2B4 cells. As shown in Figure 2A–B, we identified a total of 178 and 239 genes whose expression was significantly up- or down-regulated in infected 2B4 cells over time (i.e., 12–48 hrs), respectively. The capacity of SARS-CoV to modulate gene expression of infected 2B4 cells was not as potent as that of DHOV, whose infection resulted in 684 and 246 genes being significantly up-regulated and down-regulated, respectively (data not shown).

Among this total of SARS-CoV-regulated 417 genes (i.e., 178 up- and 239 down-regulated genes), only 8 up-regulated genes were detected at the earliest time point (i.e., 12 hrs). The expression of these genes continued to increase over time and reached the highest magnitude at 48 hrs. Functional and signaling pathway analysis of these early, virally induced and persistent host genes (N = 8) revealed that with the exception of PTX3 gene, whose function is largely associated with the regulation of innate inflammatory responses, the remaining seven genes (i.e., ATF3, EGR1, c-JUN, c-Fos, MKP-1, EGR4, and IKBα) are functionally related to transcriptional factors (TFs) by acting either as suppressors, phosphatases, or kinases (Table 1). Besides these early activated genes, there were a total of 85 and 412 genes whose expressions were significantly altered (i.e., either up- or down-regulated) at 24- and 48-hrs p.i., respectively.

**Table 1:** Common up-regulated genes in 12, 24 and 48 hrs.

Gene symbol	Name	Function	TF^a^	Fold increase		
				12hrs	24hrs	48hrs
ATF3	Activating transcription factor 3	Transcription factor in ATF/CREB family	Elk-1	1.8	2.4	3.1
MKP-1	MAP kinase phosphatase 1	Negatively regulate MAPK	Elk-1	1.6	2.3	3.0
EGR1	Early growth response 1	Transcription factor which may play roles in cell growth, apoptosis and differentiation	Elk-1, NFκB, STAT	4.2	4.9	4.7
EGR4	Early growth response 4	Autoregulatory transcriptional repressor		2.1	4.4	4.9
c-FOS	v-fos FBJ murine osteosarcoma viral oncogene homolog	Dimerise with the other AP-1 to form the AP-1 transcription factor complex	Elk-1	1.6	2.7	2.3
c-JUN	v-jun sarcoma virus 17 oncogene homolog (avian)	Dimerise with the other AP-1 to form the AP-1 transcription factor complex	Elk-1, STAT, GABP, CCAAT	1.7	2.7	3.7
IKBα	Nuclear factor of kappa light polypeptide gene enhancer in B-cells inhibitor alpha	Inhibit NFκB nuclear translocation	NFκB	1.7	3.3	5.8
PTX3	Pentraxin-related gene, rapidly induced by IL-1β	Amplification of the inflammatory reactions and regulation of innate immunity		1.8	4.4	10.6

a Enriched TF responsible for up-regulating the expression of genes at 12 hrs (see Fig. 3).

### Identification of TFs Involved in Regulating the Temporal Gene Expression of 2B4 Cells Triggered by SARS-CoV

TFs regulate gene transcription and play critical roles in various biological processes, including host innate responses against invading pathogens. The majority of TFs are also known to regulate the expression of multiple and often overlapping genes. The inferred activation of key TFs during the early phase of SARS-CoV infection (i.e., 12 hrs) (Table 1) prompted us to analyze the temporal activation of TFs by using the transcription factor database, known as TRANSFAC. Among those TFs deduced to be activated at 12 hrs, activation of NFκB, STAT, and Elk-1 persisted throughout the entire course of infection (i.e., 12–48 hrs), thereby suggesting their close role in regulating epithelial responses to SARS-CoV infection (Table 1 and Figure 3). Significant activation of other TFs belonging to either the AP-1 family (e.g., ATF2, ATF2/c-JUN, and ATF3) or the CREB/ATF family (e.g., CREB, and CREB/ATF) were also detected at 24 hrs, whereas activation of IRF-7, a molecule critically involved in the induction of type I IFNs, could not be observed until 48 hrs p.i. (Figure 3).

**Figure 3:**
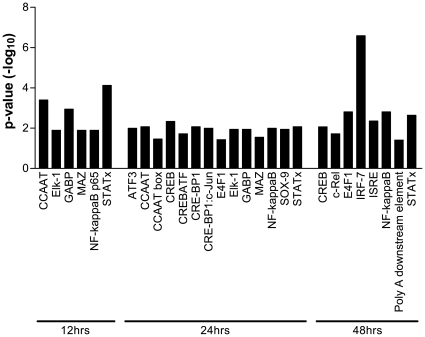
Temporal and differential activation of transcription factors (TFs) in SARS-CoV-infected 2B4 cells. Genes whose expressions were significantly altered in SARS-CoV-infected 2B4 cells were subjected to the TRANSFAC database-based analysis of TF activation. Adjusted *p* values of <0.05 among stringent pairwise comparisons were used for selecting those TFs that were significantly enriched at 12, 24, and 48 hrs p.i., respectively.

### Characterization of the Biological Functions of SARS-CoV-Altered Genes

**Figure 4:**
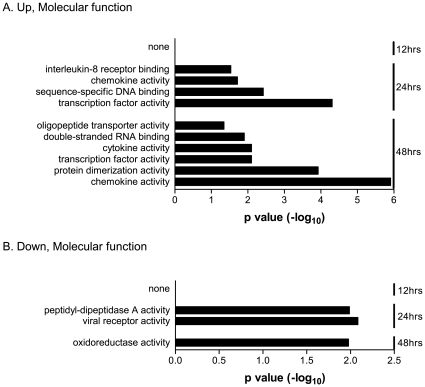
Enriched molecular functions of those genes whose expressions in 2B4 cells were significantly altered by SARS-CoV. Expressions of both up- and down-regulated genes at 12, 24, and/or 48 hrs after SARS-CoV infection were analyzed against the entire human genome gene set. The enriched GO-annotated terms identified for those up-regulated and down-regulated genes are presented in A and B, respectively. The height of an individual bar represents the level of the statistical significance of the enriched GO-annotated term. An adjusted *p* value of <0.05 was used as the criterion for selecting enriched molecular functions.

**Figure 5:**
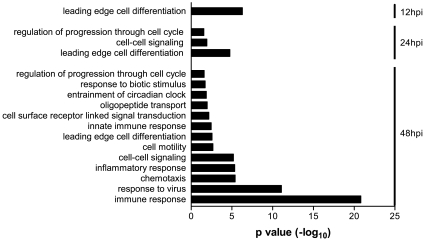
Enriched entities of biological processes for those genes that were highly activated in 2B4 cells in response to SARS-CoV infection. While no enriched GO-annotated biological term could be identified for those genes whose expressions were significantly down-regulated, 13 enriched biological entities were readily revealed for those highly activated genes of SARS-CoV-infected 2B4 cells at 12, 24, and/or 48 hrs p.i.. The height of each individual bar represents the level of statistical significance of the enriched GO-annotated biological process. An adjusted *p* value of <0.05 was used as the minimal criterion for selecting the enriched GO-annotated biological functions.

To identify functional patterns that might allow us to better understand the biological relevance of the temporally regulated genes of infected 2B4 cells, all of the significantly up- and down-regulated genes were subjected to gene ontogeny (GO)-based annotation and functional analysis. Those that were applicable, namely the enriched GO terms of genes analyzed, are depicted as Figures 4 and 5, according to their molecular function and biological process, respectively.

All of the functionally enriched GO terms identified thus far for those genes up-regulated at 24 and 48 hrs p.i. appeared to be closely involved in either genomic transcription (i.e., TFs, sequence-specific DNA and/or double-stranded RNA binding, oligopeptide transporter, and protein dimerization) or inflammatory responses (Figure 4A). The initial activation of genes encoding inflammatory cytokines and/or chemokines was detected first at 24 hrs, and their expressions were further enhanced at 48 hrs. In contrast, the activities of peptidyl-dipeptidase A, i.e., angiotensin converting enzyme (ACE) and ACE2 (i.e., SARS-CoV receptor) of infected 2B4 cells were significantly reduced at 24 hrs (Figure 4B). Because ACE2 has a protective function against acute lung injury [48], its reduced expression as the consequence of SARS-CoV infection might worsen the pathogenesis resulting from SARS-CoV infection. While the expressions of genes functionally related to oxidoreductase were also reduced at 48 hrs, their subsequent impact on the host responses and/or SARS pathogenesis remains currently unknown.

When these significantly modulated genes were analyzed, according to the biological process, we could not identify any enriched GO terms for those genes whose expressions were down-regulated over time. In contrast, we were able to assign 13 enriched GO-annotated terms that describe the “biological themes” of those genes whose expressions were up-regulated in infected 2B4 cells (Figure 5). Among these 13 annotations of the biological entities, the expressions of genes directly involved in the process of cell differentiation, cell cycle progression, and cell-to-cell signaling were significantly up-regulated as early as 12 hrs and remained highly elevated at 24 and 48 hrs p.i., whereas genes highly relevant to host immune responses were not induced until 48 hrs p.i.. Interestingly, the highly elevated expressions of genes with the GO-annotated biological functions of “response to virus” and “immune response” were particularly prominent, as supported by the extremely low *P* values.

**Figure 6:**
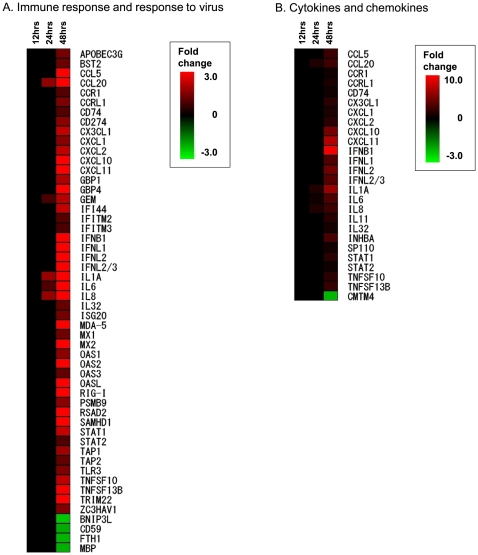
SARS-CoV-infected 2B4 cells elicit temporal and transitional expressions of genes relevant to innate immune responses. The expression of 52 and 26 genes relevant to host viral immune responses (A) and the expression of cytokines and chemokines (B) elicited by SARS-CoV-infected 2B4 cells were hierarchically clustered along with time points after infection to more comprehensively reveal the temporal and transitional nature of host antiviral and inflammatory responses to SARS-CoV infection.

It is thought that SARS pathogenesis likely stems, in part, from ill-regulated and often excessive innate inflammatory responses against respiratory SARS-CoV infection. To more relevantly measure the possible impact that infected lung epithelial cells might have on temporal host immune responses *in vivo*, we generated heat maps of two elevated gene sets of infected 2B4 cells, representing immune responses/response to virus (N = 52), and cytokines/chemokines (N = 26), respectively. As shown in Figure 6A–B, genes encoding CCL20, IL-1A, IL-6, and IL-8 appeared to be among the first batch of inflammatory genes activated by SARS-CoV at 24 hrs. The induction of these early inflammatory genes continued to increase at 48 hrs, accompanied by the up-regulation of other host genes relevant to innate inflammatory and/or antiviral processes, including CXCL10, CXCL11, IFN-β1, IFN-λ1, - λ 2, - λ 3 and many interferon-stimulated genes (ISGs), such as ISG20, MXs, OASs, RIG-I, MDA-5, TLR3, STATs, etc..

### Confirmation of the Elevated Transcription of IL-6-, IL-8-, CXCL10/IP-10-, CCL5/RANTES-, and IFN-λ-Encoded Genes

**Figure 7:**
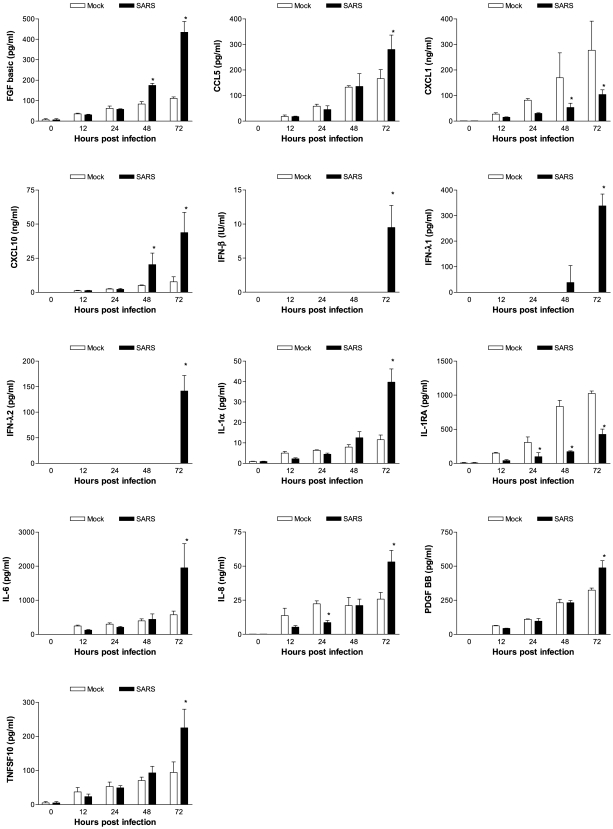
Confirmation of the transcription of pro-inflammatory genes at the translational level. Cell-free supernatants harvested from mock- and SARS-CoV-infected 2B4 cultures at indicated time points p.i. were assessed for the concentration of various inflammatory mediators by using the human group I 27-plex and group II 8-plex Cytometric Bead Array, ELISA (for IFN-β and –λs), as described in Materials and Methods. *, p<0.05 by two-way ANOVA with Bonferroni's post-hoc test in comparison with the mock-infected group at the same hrs p.i.

To determine whether transcriptional activation of genes coding for various inflammatory mediators, especially IFN-β and IFN-λs, could be extended to the translational levels, gamma (γ)-irradiated supernatants of mock-infected and SARS-CoV-infected 2B4 cultures (MOI = 0.1) at indicated time points p.i. were subjected to Bio-Plex, ELISA, and/or VSV/Vero-based plaque-reduction assays for assessing the contents of cytokines, chemokines, and IFNs. Among 35 cytokines and chemokines that could be simultaneously measured by using the human group I and II Bio-Plex, we were able to detect significantly elevated expression of fibroblast growth factor (FGF) basic, CCL5/RANTES, CXCL1/GROα, CXCL10/interferon-inducible protein (IP)-10, IL-1α, IL-6, IL-8, platelet-derived growth factor (PDGF) BB, and TNFSF10/TRAIL at either 48 or 72 hrs p.i. (Figure 7). We could also detect the increased expression of IFN-β, IFN-λ1, and IFN-λ2 proteins at 72 hrs by ELISA. While the induction of many host genes could be confirmed at both transcriptional and translational levels, the enhanced expression of genes coding for FGF basic and PDGF BB, two molecules frequently implicated as participating in the process of various pathological conditions, could only be detected at the translational, but not transcriptional, levels at 48 and/or 72 hrs p.i. Contrary to the elevated expression of most inflammatory mediators, the production of IL-1RA, an antagonist of IL-1α and -1β, by infected 2B4 cells was significantly reduced as early as 24 hrs p.i. and sustained through 72 hrs p.i..

### Expression Correlation between mRNAs and Their Corresponding Proteins in 2B4 Cells

**Figure 8:**
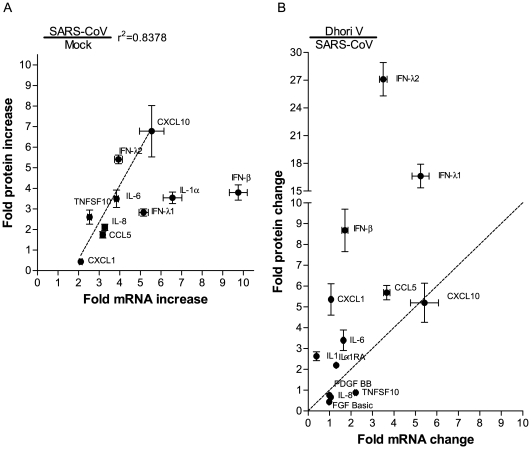
Correlation between transcript and protein levels of genes encoding chemokines, cytokines and IFNs. The relative transcriptional-versus-translational efficacies of selected genes in 2B4 cells were assessed, based on the fold-increase of mRNA and the corresponding protein levels of infected cells over those of mock-infected ones. A strong correlation between transcriptional and translational expressions of genes coding for CCL5/RANTES, CXCL1/GROα, CXCL10/IP-10, IFN-λ2, IL-6, IL-8, and TNFSF10/TRAIL (r^2^ = 0.8378) was identified in SARS-CoV-infected 2B4 cells. Dotted line indicates regression line (A). The relative efficacy of gene expression among those highly activated in SARS-CoV-infected and DHOV-infected 2B4 cells was compared as described in a subsection of Results. Dotted line (y = x) separates the genes to verify which virus (DOHV; upper part, SARS-CoV; lower part) -infected 2B4 cells effectively translate the genes than the other virus-infected one. It became clear that translational expressions of CCL5/RANTES, CXCL1/GROα, IL-1α, IL-6, and, particularly of IFN-β, IFN-λ1 and –λ2 were efficient in DHOV-infected 2B4 cells. The efficacy of CXCL-10/IP-10, IL-8 and TNFSF10/TRAIL expression was similar between these two differentially infected 2B4 cells (B). Results are shown as mean ± SEM for nine calculated results by division of each triplicated samples in indicated cytokines.

The discordant mRNA and protein expression of genes encoding for FGF basic, PDGF BB, and, IL-1RA in SARS-CoV-infected 2B4 cells prompted us to evaluate the correlativity of mRNA and the corresponding protein expression among a cohort of virally activated genes, including CCL5/RANTES, CXCL1/GROα, CXCL10/IP-10, IFN-β, IFN-λ1,IFN-λ2, IL-1α, IL-6, IL-8, and TNFSF10/TRAIL. We selected 48 hrs and 72 hrs p.i., two of the most conspicuous time points for detecting up-regulated mRNA and protein levels, respectively, for this analysis. We calculated the fold-increase of either mRNA or the protein of individual genes elicited by infected 2B4 cells over those that were mock-infected (N = 3) at the indicated time points by simply dividing the raw values of mRNA or protein for individual genes expressed in infected cells by those detected in mock-infected ones. The resulting values were subjected to the Spearman correlation coefficient analysis for establishing the interrelationship between mRNA and protein expression of targeted genes. As shown in Figure 8A, there existed a very strong and positive correlation between the transcriptional and translational expressions of genes encoding CXCL10/IP-10, IFN-λ1, IL-6, TNFSF10, CCL5/RANTES, IL-8, and CXCL1, respectively (r^2^ = 0.8378, *p*<0.01). In contrast, the levels of mRNAs and their corresponding proteins correlated poorly for IFN-λ2-, IL-1α-, and IFN-β-encoding genes, in that the levels of mRNA transcripts were more readily detectable than those of the proteins.

To determine whether such poor efficiency in protein expression of IL-1α, IFN-λ2, and, particularly, of IFN-β by 2B4 cells, was a unique consequence of SARS-CoV infection, we performed a parallel analysis of the mRNA and protein expression profiles of those genes whose expressions were significantly activated in 2B4 cells upon infection with SARS-CoV or DHOV by comparing the expression levels of mRNA and protein of the targeted genes. Briefly, the relative mRNA and protein expression efficacies of genes elicited by differentially infected 2B4 cells were calculated by dividing the amounts of mRNA or protein detected in SARS-CoV-infected cultures by those expressed by DHOV-infected ones harvested at the same time points p.i.. As shown in Figure 8B, we drew a dotted line representing a “one-to-one” ratio of the gene mRNA and protein expression. Thus, genes plotted above this dotted line would represent those whose protein expression after mRNA transcription were more efficiently expressed by DHOV-infected cells, whereas genes plotted underneath the dotted lines represented those whose expression was more readily detectable by SARS-CoV-infected cultures. It became clear that while SARS-CoV-infected 2B4 cells were able to retain their ability to express some of the activated genes, such as TNFSF10, IL-8, and, especially, CXCL10/IP-10, they were particularly inefficient in the post-transcriptional expression of many antiviral genes, especially those encoding for IFN-β, IFN-λ1, and IFN-λ2, when compared to DHOV-infected cells. Taken together, our results lead us to suggest that SARS-CoV might have evolved strategies, likely via preferentially targeting IFN-related antiviral genes at post-transcription level, to successfully establish infection in the immune-competent 2B4 cells.

### Synergistic Effect of IFN-β and IFN-λs Against SARS-CoV Replication

**Figure 9:**
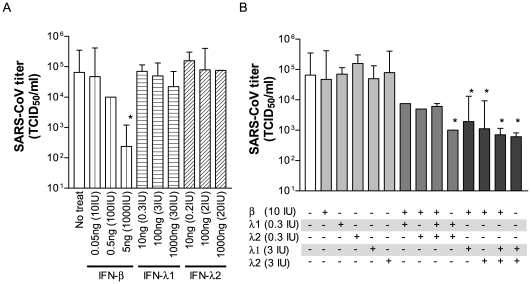
Efficacy of IFN-λs in the host defense against SARS-CoV infection. Confluent cultures of 2B4 cells were treated with rIFN-β, rIFN-λ1, or rIFN-λ2 at the indicated concentrations alone (A) or in combination (B) for 24 hrs prior to their infection with SARS-CoV (MOI = 0.01). Resulting supernatants were harvested at day 2 after infection for assessing yields of infectious progeny virus by the standard Vero E6-based infectivity assay. *, p<0.05 by a two-way ANOVA with Bonferroni's post-hoc test in comparison with IFN-untreated cultures. Data shown were representative of two independent experiments.

Type I IFNs (IFN-α/β) rapidly produced by the infected host serves as the first line of defense against viral infections, in part, via triggering the expressions of many ISGs to combat the invading pathogens. The ability of SARS-CoV to activate MXs, OAS, RIG-I, MDA5, TLR3, STATs, and many other ISGs without stimulating any significant responses of IFN-β, and especially of IFN-αs, prompted us to investigate whether IFN-λs elicited by infected 2B4 cells could potentiate IFN-α/β-like activity against SARS-CoV. To evaluate the potential of IFN-λs alone or in combination with type I IFNs against SARS-CoV infection, we pretreated 2B4 cells with IFN-β, IFN-λ1 and IFN-λ2, either individually or in combination at indicated concentrations prior to infection with SARS-CoV (MOI = 0.01). While IFN-β at the concentration of 5 ng (equivalent to 1,000 IU) alone had a significant antiviral effect, we were unable to reveal any antiviral effect of either IFN-λ1 or -λ2 when used alone at a concentration as high as 1,000 ng (Figure 9A). However, a combinational treatment of both IFN-λ1 and IFN-λ2, even at a concentration as low as 10 ng each, significantly reduced SARS-CoV replication (*P*<0.05), which suggested to us that both types of IFN-λs are required to effectively limit the replication of SARS-CoV (Figure 9B). Additionally, treatment with either type of IFN-λ, together with an otherwise ineffective low-dose of IFN-β (e.g., 10 IU), drastically hampered the growth of SARS-CoV in 2B4 cells (*P*<0.05), a finding which may imply that either species of IFN-λ could potentiate the antiviral effect of IFN-β. Surprisingly, we also found that pretreatment of cells with all three types of IFNs together (i.e., IFN-λ1, IFN–λ2, and IFN-β) at a low-dose regimen (i.e., 10 ng each for IFN-λ1 and –λ2 and 0.05 ng for IFN-β) diminished the antiviral effect provided by the combination treatments of IFN-λ1/IFN-β, IFN-λ2/IFN-β, and IFN-λ1/IFN-λ2 at the same concentrations. While the mechanism of this interesting observation remains unknown, our results showed that IFN-λs possess a previously unidentified type I IFN-like activity against SARS-CoV.

## Discussion

Intense acute inflammatory responses, concomitant with extremely unremarkable IFN-α/β secretion, are the hallmark of SARS-CoV infection. However, the molecular mechanisms attributed to such highly dysregulated innate antiviral responses in SARS-CoV-infected cells, especially those of pathologically relevant lung epithelial cells, remain elusive. We have reported that whereas human bronchial Calu-3 cells secreted high levels of biologically active IL-6, IL-8, and IP-10 in response to SARS-CoV infection, they failed to mount any detectable IFN-α/β response [38]. Because Calu-3 cells are highly heterogeneous with regard to the expression of the ACE2 viral receptor [41], we strived and succeeded to establish various clones of Calu-3 cells, which would be valuable for studying the host response to SARS-CoV infection.

The global host gene expression in response to SARS-CoV infection has been previously reported in various cell types, including the less permissive (if any) human PBMC [14], [18] and primary MΦ [19], highly permissive, but pathologically irrelevant Caco-2 cells [49] and Huh7 cells [50] and even Vero E6 cells [21]. In addition, the elucidation of the host gene expression of most of these studies has been based on a single time point after infection, ranging from 2 to 24 hrs p.i., with the study of Huh7 cells being the only exception in which RNA samples collected at both 2 and 4 hrs p.i. were analyzed. Based on their stable and intense ACE expression and, thus, enhanced permissiveness to productive SARS-CoV infection, compared to that of parental Calu-3 cells (Figure 1), we chose the cloned 2B4 cells for studying the temporal expression of airway epithelial genes related to the innate antiviral defense against SARS-CoV infection by using microarray-based functional genomics.

It has been well documented that innate antiviral signaling is initiated upon the recognition of conserved molecular structures shared by invading pathogens of various origin, known as pathogen-associated molecular patterns (PAMPs), by specific pattern recognition receptor (PRR) molecules expressed on host cells, which ultimately leads to the activation of transcriptional factors, primarily IRF3/7 and NFκB, for the induction of IFN-α/β and other proinflammatory mediators [51], [52], [53]. By using this *in vitro* system, we demonstrated that NFκB and STAT were among the earliest TFs activated at 12 hrs. The fact that their activation persisted throughout the entire course of infection (i.e., 12 to 48 hrs) (Figure 3) strongly argues for their important role in regulating epithelial responses to SARS-CoV. The critical roles of NFκB- and STAT-mediated signaling pathways in the activation of many ISGs and genes coding for various pro-inflammatory mediators have been well characterized [54], [55]. Although several other TFs belonging to the AP-1 and CREB/ATF families and their heterodimeric complexes, i.e., ATF3, CREB, CREBATF, CRE-BP1, and CRE-BP1-c-JUN, all of which are known to regulate the transduction of many inflammatory genes, were subsequently activated at 24 hrs, the activation of IRF-7 did not occur until 48 hrs p.i. (Figure 3). Such a delay in the activation of IRF-7 relative to that of NFκB and AP-1 in 2B4 cells appeared to be unique to SARS-CoV as it was not observed in DHOV-infected 2B4 cells (data not shown).

The ability to rapidly synthesize and secrete type I IFNs, especially IFN-β, is a key element of the innate antiviral responses. Effective transcriptional induction of the IFN-β gene requires coordinated induction and binding of at least three promoter-specific TFs (i.e., NFκB, IRF-3, and ATF-2/c-Jun) [56], [57]. Although activation of NFκB- and/or AP-1 (c-JUN/ATF2)-mediated pathways could contribute to IFN-β gene induction [58], studies with targeted gene knockout (KO) mice have clearly indicated that IRF-3 and/or IRF-7 molecules play an indispensible, but distinctive, role in the induction of IFN-α/β responses against viral infection [59]. IRF-3 is constitutively expressed in many cells, whereas the expression of IRF-7 is highly inducible by IFNs [60], [61]. It has been demonstrated that activation of IRF-3 is primarily responsible for the early and limited induction of IFN-α/β genes, which, in turn, bind to their corresponding type I IFN receptors in an autocrine and/or paracrine manner to initiate a positive feedback loop through the induction and subsequent activation of IRF-7 for furthering IFN-α/β production [62]. Unfortunately, the TRANSFAC database that we used in this study to identify enriched TFs did not contain data on IRF-3 or its regulatory binding sites, preventing us from deducing whether IRF-3 activation was induced in 2B4 cells upon SARS-CoV infection. However, the elevated transcriptional activation of genes coding for IRF-7, IFN-β, IFN-λs, and many ISGs detected at 48 hrs in infected 2B4 cells strongly argue for the activation of IRF-3 by SARS-CoV in 2B4 cells. However, the impact of such a delay in the activation of the indispensible IRF-3/7 pathways, relative to those of NFκB and/or ATF2/c-Jun, in the induction of potent, IFN-associated epithelial antiviral responses might be of significance.

The molecular mechanism responsible for the delay of IRF-3/7 activation in infected 2B4 cells remains unknown. However, it has been reported that SARS-CoV is capable of evading innate antiviral responses by encoding antagonistic molecules that target different host antiviral pathways. Specifically, ORF3b, ORF6, and N proteins of SARS-CoV can counteract host innate antiviral responses by specifically inhibiting the activation of IRF-3, leading to the inhibition of IFN-β gene induction [29], whereas SARS-CoV-encoded ORF7 and nsp1 proteins can facilitate immune evasion by either promoting degradation or inhibiting translation of cellular mRNA transcripts [31], [63], [64]. It was also reported that SARS-CoV could directly interrupt the nuclear translocation of activated STAT1 to prevent the induction of IFN-mediated antiviral responses [32], [65]. Finally, the ability of SARS-CoV M protein to actively interfere with the formation of the TRAF3.TANK.TBK1/IKKε complex, thereby inhibiting TBK1/IKKε-dependent activation of IRF3/IRF7, is yet another mechanism by which SARS-CoV prevents the production of IFN-α/β [27]. Taken together, SARS-CoV appeared to preferentially target the TBK1/IKKε-IRF-3/7-STAT axis of the IFN-related signaling pathway, rather than inflammatory response-bound NFκB- and/or ATF2/c-Jun pathways, to circumvent the host innate antiviral activities. However, such interferences in the TBK1/IKKε-IRF-3/7-STAT- signaling axis imposed by SARS-CoV in 2B4 cells appeared to be incomplete, as evidenced by the increased expression of many antiviral genes, such as IFN-β, IFN-λs, and ISGs, at 48 hrs (Figure 6A–B). Thus, like many other viruses, SARS-CoV might have established strategies to antagonize host antiviral responses by delaying, rather than completely blocking, IFN-related IRF-3/STAT1-mediated signaling pathways [66].

The early and enhanced expression of CXCL-10/IP-10 (Figures 7 and 8), a potent chemoattractant for activated T cells and NK cells [67], [68] within the circulation and the lungs of patients affected by SARS, has been associated with adverse outcomes in SARS [11], [12]. Thus, the ability of infected 2B4 cells to retain a prominent and highly correlated transcriptional and translational activation of the CXCL-10/IP-10 gene, but not others (Figures 7 and 8), might be relevant to SARS pathogenesis. In contrast, such a highly correlated mRNA-protein expression was not observed for IFN-λ2 and, especially, IFN-β. Specifically, the expressions of IFN-λ2 and IFN-β genes in SARS-CoV-infected 2B4 cells were more readily detected at the transcriptional rather than the translational levels (Figure 8A). Importantly, such disparities of the post-transcription efficiency among genes tested in this study were not inherited by 2B4 cells, as cells infected by DHOV were quite capable of expressing transcripts and proteins of IFN-λ2 and IFN-β in a highly correlated manner (Figure 8B and Hill, T. et al, in preparation). Taken together, these results suggest that while the expressions of IFN-λ2 and, especially of IFN-β proteins, were profoundly inhibited, SARS-CoV infection did not impose a generalized suppression of host posttranscriptional machinery in 2B4 cells, an observation consistent with that reported for SARS-CoV nsp-1 protein [12], [64]. The failure of effectively translating IFN-β transcripts has also been reported recently in mouse fibroblasts infected by MHV stain A59. Interestingly, such an inhibition of the production of IFN-β protein appeared to occur at the post-transcriptional level without affecting the stability of either mRNA transcripts or synthesized protein [69].

With the exception of CXCL1 and CXCL10/IP-10, enhanced protein levels of most of the aforementioned genes, including IFN-β and IFN-λs, could not be convincingly detected until 72 hrs p.i. (Figure 7); however, transcripts of many ISGs (e.g., MXs, OASs, RIG-I, MDA-5, TLR3, STATs, and ISG20) were present at 48 hrs, thereby strongly arguing for the likelihood of a minute, but physiologically relevant, amount of IFN-β and/or IFN-λ proteins, which was otherwise undetectable by the Bio-Plex, ELISA, and/or the standard plaque reduction assay, being produced by SARS-CoV-infected 2B4 cells early after infection.

Similar to the induction of IFN-α/β expression, virally induced expression of IFN-λs also relies on the activation of the RIG-I-MAVS/IPS-1-TBK1/IKKε-IRF3 signaling axis [70]. Despite such striking similarities between these two classes of IFNs with regard to their patterns of activation, expression, and biological functions, it was recently reported that the virally induced production of IFN-λs was much more intense in the stomach, intestines, and lungs, when compared to findings in the CNS and spleen. Furthermore, epithelial cells seemed to be preferentially responsive to IFN-λs, when the latter results were compared to those from other cell types, thereby leading to the suggestion that IFN-λs exhibit some tissue and cell specificity [71]. Recent studies that compared the role of IFN-α/β and IFN-λs in the host innate immunity against respiratory influenza A virus versus hepatotropic Thogotovirus in IFN receptor knockout mice have indicated that IFN-λs contribute to the host defense viral pathogens infecting the lung but not the liver, further emphasizing a tissue-restricted manner of IFN-λ-mediated antiviral responses [72]. Thus, the use of human bronchial epithelial 2B4 cells may be responsible for our success in identifying highly elevated expressions of IFN-λ genes as a novel biomarker of SARS-CoV infection.

While the efficacy of IFN-α/β in prophylaxis against SARS-CoV infection has been well established, the relative contribution of IFN-λs in the epithelial defense against SARS-CoV infection has yet to be explored. Here, we showed that SARS-CoV replication could be drastically inhibited by IFN-λs (i.e., IFN-λ1 and -λ2), when used together, even at a low dose (10 ng per each of λ1, ∼0.3 IU and λ2 ∼0.2 IU), but not when either IFN was used alone, even at a high dose (1,000 ng per each of λ1 ∼30 IU and λ2 ∼20 IU), to treat 2B4 cells for 24 hrs before viral challenge (MOI = 0.01). Furthermore, the combined IFNλs inhibited the replication by a factor of ∼100 to a level comparable to that exerted by 5 ng (∼1,000 IU) of IFN-β. Furthermore, either type of IFN-λs, when used at a concentration of 100 ng (i.e., ∼3 and 2 IU of λ1 and λ2, respectively), but not at 10 ng, when combined with an otherwise ineffective dose of IFN-β (i.e., 0.05 ng/10 IU), could highly potentiate antiviral responses, resulting in the reduction of SARS-CoV replication by a factor of ∼50–75 at day 2 after infection (Figure 9). While the efficacy of IFN-λs against SARS-CoV replication, when used alone or in combination with IFN-β, has been consistently observed in our studies, additional *in vitro* and animal studies are warranted to determine the mechanism(s) of protection and whether type I and type III IFNs exert a redundant, complementary, or synergistic role in the host defense against SARS-CoV infection.

In summary, we demonstrated that human bronchial epithelial 2B4 cells are capable of responding to SARS-CoV infection by temporally activating latent NFκB (12-hrs), AP-1 (ATF2/-Jun) (24-hrs), and IRF-3/7 (48-hrs), all of which, especially IRF-3/7, are critically involved in the induction of type I IFN gene expression and the subsequent activation of virus-dependent and/or IFN-dependent antiviral signaling pathways. Early activation of NFκB and AP-1 in epithelial cells would lead to the production of inflammatory mediators, especially IL-6 and IL-8, which have the potential to exert detrimental effects on the host immune response, in part, by modulating the intrinsic functions of resident MΦ and DCs [38]. Furthermore, because IRF-3/7 play an indispensible role for the induction of the optimal expression of type I IFNs, their delayed activation, relative to NFκB and AP-1, would likely postpone the onset of type I IFN-mediated innate antiviral responses, thereby greatly compromising the ability of the infected host to keep early viral replication in check. Despite their delayed expressions, we demonstrate, for the first time, that both IFN-α/β and IFN-λs possess non-redundant and, therefore, complementary activities against SAS-CoV replication. We also identify various ISGs elicited by lung epithelial cells, the primary target cells of SARS-CoV infection, which might be crucial targets for not only better understanding the initiation of innate and adaptive immunity, but also for the future development of innovative therapeutics against SARS.

## Materials and Methods

### Cells

Vero E6 cells [CRL-1580, American Type Culture Collection (ATCC)] were grown in Eagle's minimal essential medium (MEM) supplemented with 10% fetal calf serum (FCS), designated M-10 medium, whereas human bronchial epithelial Calu-3 cells (HTB-55, ATCC) were grown in MEM medium supplemented with 20% FCS (M-20) medium. Calu-3 cells, which originated from a human pulmonary adenocarcinoma, have been well characterized as non-ciliated human bronchial epithelial cells with a mixed phenotype and have been used in many areas of biomedical research since their establishment.

### Viruses

The Urbani strain of SARS-CoV, kindly provided to us by Dr. T. G. Ksiazek at the Center for Disease Control and Prevention (Atlanta, GA), was used throughout this study. The original stock of SARS-CoV was subjected to two additional passages in Vero E6 cells. A viral stock with a titer of 1×10^7^ 50% tissue culture infectious doses (TCID_50_)/ml was generated and stored at −80°C.

The prototype strain (IG 611313) of Dhori virus (DHOV), an orthomyxovirus, was also used in this study. Although DHOV was originally isolated from *Hyalomma dromedarii* ticks [73], intranasal infection of mice with this tick-borne virus resulted in the onset of a fulminant and uniformly fatal illness with many of the clinical, inflammatory, and pathologic features highly resembling to those observed in mice infected with H5N1 highly pathogenic avian influenza A virus (i.e., H5N1) [45], [46]. The original stock of DHOV, provided by Dr. Bob Tesh (Department of Pathology, UTMB), was subjected to three additional passages in Vero E6 cells before being used in this study.

All experiments involving infectious DHOV and SARS-CoV were conducted at UTMB in approved biosafety level-2 and -3 laboratories, respectively.

### Establishing and Characterizing the Clonal Derivatives of Calu-3 Cells

To establish Calu-3 cell populations homogeneously expressing ACE2 at a given intensity, we performed standard limiting dilution assays. Briefly, the single-cell suspension of the parental Calu-3 cells was subjected to serial 10-fold dilutions with M-20 medium, followed by cultivation in 96-well microtiter plates (1 cell/well) without the addition of feeder cells. Confluent subcultures were transferred to 24-, 12-, and 6-well plates before further expansion into culture flasks. Individual clones were subjected to analyses for the expression of ACE2 and their susceptibility to SARS-CoV infection by immunohistochemistry (IHC) and Vero E6-based infectivity assays, respectively.

### Infectious Center Assay

The infectious center assay, as described by Dutta and Myrup [74], was used to estimate the percentage of SARS-CoV-infected 2B4 cells over time. Briefly, SARS-CoV-infected 2B4 cells (MOI = 0.1) grown on the 24-well plates were washed with phosphate-buffered saline (PBS) at indicated time points after infection to remove the cell-free viruses. The infected 2B4 cells were harvested by adding Trypsin-EDTA, and next total cell recovery was counted. Cells were further washed (2×) with M-10 media, and the final washing was saved for determining the titer of the residual “cell-free” viruses by the standard plaque forming assay, as the control. The fixed numbers of “infected” 2B4 cells were then transferred to confluent Vero E6 monolayers grown in 12-well plates. After incubation for ∼30 min, the “infected” Vero E6 monolayers were overlaid with MEM medium supplemented with methylcellulose (1%) and FCS (2%) for three days before fixing and staining by paraformaldehyde (4%) and crystal violet (2%), respectively, to enumerate the plaques for determining the percentage of infected cells at individual time points.

### Western Blotting

Confluent Calu-3 and 2B4 cells grown onto 6-well plates were lysed by the standard buffer containing proteinase inhibitor. After removing cell debris by centrifugation, the total protein concentrations of the supernatants were measured by using a BCA protein assay kit (Thermo Fisher Scientific, Rockford, IL). The protein samples (20 µg each) were mixed with 2× Novex® Tris-Glycine SDS sample buffer (Invitrogen, Carlsbad, CA), heat-inactivated, fractionated by SDS-polyacrylamide gel electrophoresis, and subjected to standard Western blot transfer to a membrane. The membrane was tehn incubated with 2% blocking reagent supplemented in the ECL Advance western blotting detection kit (GE Healthcare, Piscataway, NJ) overnight, followed by additional incubation overnight with either goat anti-human ACE2 polyclonal antibody (0.1 µg/ml) at 4°C or mouse anti-β-actin (1∶1250; Cat #: ab6276, Abcam, Cambridge, MA). After it was washed with TBS (3×), the blotted membrane was allowed to react for another 2 hours with an HRP-conjugated rabbit anti-goat IgG (0.002 µg/ml; Cat #: sc-2768, Santa Cruz Biotechnology, Santa Cruz, CA) or HRP-conjugated goat anti-mouse IgG (0.0025 µg/ml; Cat #: ab5879, Abcam). After washing with TBS (3×), immunoreactive proteins were visualized by exposing them to X-ray film by using the ECL Advance western blotting detection kit according to the protocols provided by the manufacturer.

### Transmission Electron Microscopy (TEM)

Highly polarized 2B4 cells grown onto the membrane inserts under an air-liquid phase were fixed with a mixture containing 2.5% formaldehyde, 0.1% glutaraldehyde, 0.03% trinitrophenol, and 0.03% CaCl2 in 0.05 M cacodylate buffer (pH 7.2) for at least 2 hrs. After washing with 0.1 M cacodylate buffer, the cells were fixed with 1% OsO4 in the same buffer, stained with 1% uranyl acetate in 0.1 M maleate buffer (pH 5.2), dehydrated in ethanol, and embedded in Poly/Bed 812 (Polysciences, Warrington, PA). Before embedding, membranes were cut out, sliced into stripes, and stacked at one side of a flat embedding mold. Semi-thin sections were cut first, stained with toluidine blue, and examined to choose the areas of confluent monolayers for the subsequent ultra-thin sectioning by using a Leica-Reichert Ultracut S ultramicrotome. After they were stained with 2% aqueous uranyl acetate and lead citrate, the sections were examined under a Philips 201 or CM-100 electron microscope at 60 kV.

### Immunohistochemistry (IHC)

We employed the standard IHC for documenting the expression of ACE2 and SARS-CoV infection. Briefly, SARS-CoV-infected Calu-3 cultures grown on 24-well plates were washed with phosphate-buffered saline (PBS), fixed in 4% paraformaldehyde overnight, and rinsed (2×) with PBS. After blocking with PBS containing 5% bovine serum albumin for 30 min, Calu-3 cells were incubated for 1 h at 37°C with rabbit anti-SARS-CoV nucleocapsid protein (NP) (i.e., 2 µg/ml; Cat. #: IMG-548, IMGENEX, San Diego, CA). After washing (3×) with PBS, cells were stained with diluted (1∶100), biotinylated goat anti-rabbit IgG (DAKO, Glostrup, Denmark) at 37°C for an hour. Visualization of NP was achieved by incubation with streptavidin-alkaline phosphatase and naphthol-fast red substrate (DAKO).

### Microarray Analysis

DNA microarray experiments were performed in the Molecular Genomics (MG) Core Facilities at UTMB. Detailed information about the procedures used has been posted on the MG Core Facilities website (genomics@scms.utmb.edu). To characterize the dynamic, spatial, and temporal changes of gene expression induced by SARS-CoV or DHOV, confluent 2B4 cells grown in T-75 flasks were infected with either viruses (MOI = 0.1) or remained uninfected (as a control) for 12, 24, and 48 hrs. To meet the minimal number required for application of statistical algorithms, we performed the study in triplicate at each time point for mock-, and virally infected cultures, yielding a total of 27 arrays.

Briefly, supernatants were harvested from differentially treated cultures at 12, 24, and 48 hrs p.i. for subsequent analyses of viral yields and cytokine profiling, whereas the cells were subjected to total RNA extraction by using an RNAqueous-4PCR kit and following the protocol recommended by the manufacturer (Ambion, Austin, TX). Although we did not perform this microarray-based analysis on infected cells after 48 hrs p.i., supernatants of parallel 2B4 cultures infected with SARS-CoV or DHOV for 72 hrs were harvested for profiling the inflammatory responses. Purified RNA samples were sent to our core facility for conversion to cDNA, biotin-labeled, and hybridized to 27 Affymetrix Human Genome U133 Plus 2.0 “Gene Chips” each of which contained 54,675 probe set identifiers representing more than ∼47,400 transcripts that identify ∼38,500 well-characterized genes, and various internal controls (Affymetrix, Santa Clara, CA). Mock-infected cells were compared to cells infected with either SARS-CoV or DHOV at each time point. For comparison across different arrays, the data for each array were normalized by Robust Multi-chip Average (RMA) [75] using Spotfire DecisionSite (Spotfire, Inc., Somerville, MA) and GeneSifter (VizX Labs, Seattle, WA), and probe sets with expression values below the level of background noise (as determined by detection *p* value) were disregarded in further analyses. Results of these analyses indicated that the RNA samples used and the data generated after normalization were of sufficiently high quality for subsequent analyses (data not shown).

For statistical purposes, all 27 arrays were analyzed as 18 separate groups (mock-, SARS-CoV-infected, and DHOV-infected cells at 12, 24, and 48 hrs). The GeneSifter-based normalization was followed by pairwise comparisons of average group values and Student's *t* test with Benjamini and Hochberg correction [76]. Criteria for the selection of genes were as follows: All pair-wise comparisons for one group versus another (e.g., mock-versus-SARS-CoV or DHOV-infected cells at 12 hr) were expected to be at least 1.5-fold and at least 50% greater than the fold-change observed between any two controls (e.g., mock-infected replicate 1 versus mock-infected replicate 2 at 12 hr). Exceptions were made for those probe sets that were significantly altered (fold change ≥1.5, p value<0.05) at an earlier or later time point, with a magnitude that exceeded the observed fold-change between replicate controls by at least 50%. This allowed for more accurate identification of those genes that increased or decreased in expression levels over time but that fell below the stringent statistical criteria at the lower transcriptional levels. In order to ensure that results obtained from comparison of virally infected cells were specific to infection, any probe set determined to be significantly different in hybridization signal based on the above criteria was further expected to have been detected as altered (fold-change ≥1.5 and at least 50% greater in magnitude than any change observed between control samples, p value ≤0.05) between mock-infected and virally infected cells. In other words, any gene with an expression level that differed between cells infected with the two different viruses was considered as irrelevant if not altered by either virus, as determined by comparison to mock-infected control cells at that same time point.

### Data-Mining, Gene Ontology and TF Analysis for DNA Microarray Data

To identify the independence and/or redundancy of the gene expression over time (i.e., 12-, 24-, and 48-hrs p.i.) and at each time point, we produced Venn diagrams at Venny website (http://bioinfogp.cnb.csic.es/tools/venny/index.html). For functional profiling of selected genes, we employed FatiGo (http://www.babelomics.org/), which is a web-based functional analysis tool to determine whether any given cluster of genes has an enriched biologically relevant term, when compared to other gene clusters [77], [78]. Specifically, all of the genes whose expressions were significantly altered (i.e., both up- and down-regulated genes) in infected 2B4 cells at each time point, when compared to those of mock-infected cells, were analyzed against the available human genomes to assign the most characteristic gene ontology (GO) terms, such as the biological process and the molecular function, to each cluster of genes. In addition, the TRANSFAC database (http://www.gene-regulation.com/pub/databases.html) was employed for exploring transcription factors (TFs), their DNA-binding sites, and regulated genes. For the statistical analysis, we used Fisher's exact test for comparing two groups of genes for extracting the GO terms whose distribution among the groups was significantly different. All of the *p* values obtained for the selected GO terms were corrected to obtain an adjusted *p* value, according to the Benjamini and Hochberg correction [76]. Adjusted p values <0.05 were chosen to determine the level of statistical significance.

### Profiling of Cytokine and IFN Proteins Secreted by SARS-CoV-Infected 2B4 Cells

The biological activities of IFNs in culture supernatants were assessed by the standard Vero E6/vesicular stomatitis virus (VSV)-based plaque-reduction assay, as described by Langford and colleagues [79]. Briefly, supernatants of virally infected cultures were subjected to a serially 3-fold dilution on Vero E6 monolayer grown on to the 96-well plates. After incubation for 24-hrs at 37°C, the cultures were washed (3×) with phosphate buffered saline (PBS) and challenged with 50 plaque-forming units (pfu) of VSV. After absorption for an hour at 37°C, the medium was decanted to remove unbound VSV, followed by addition of 100 µl/well of culture medium containing 1% methylcellulose. The infected cultures were incubated for 24 hrs and subsequently stained with crystal violet (1%) for enumerating the plaques. Recombinant (r) IFN-β containing a specific activity of 10 IU (International Unit) per 0.05 ng of protein (The PBL Biomedical laboratories, Piscataway, NJ) was included in this IFN bioassay as the standard. The titers of IFN in the test samples were expressed as the highest dilution giving at least 50% reduction of 50 pfu of VSV. In addition, we also titrated the contents of IFN-β and IFN-λs in the supernatants by using the commercially available ELISA kits for human IFN-β and IL-28A/IFN-λ2, according to the protocols provided by the manufacturer (PBL, Piscataway, NJ). For profiling the cytokine responses of infected 2B4 cells, we employed the Bio-Plex Cytometric Bead Array (BioRad, Hercules, CA), as we previously described [38], in which both the human group I 27-plex and the group II 8-plex (i.e., CXCL1/GROα, CXCL9/MIG, IFN-α2, IL-1α, IL-12 (p40), IL-18, TNF-β, TNFSF10/TRAIL) were used.

### Evaluation of the Protective Efficacy of IFN-β and/or IFN-λs Against SARS-CoV Infection

Confluent 2B4 cells were pre-treated with rIFN-β (PBL), rIFN-λ1, or IFN-λ2 (Peprotech Inc., Rocky Hill, NJ) at indicated concentrations either alone or in combination for 24 hrs before challenging with SARS-CoV (MOI = 0.01) for 72 hrs. The resulting supernatants were harvested for assessing the yields of the infectious progeny virus by the standard Vero E6-based infectivity assay for assessing their potential in inhibiting SARS-CoV replication.

### Statistical Analysis

The results relative to cytokines, chemokines, IFN production and virus titer are presented as the mean ± standard deviation (SD). In addition, a two-way ANOVA with Bonferroni's post-hoc test was used to establish statistical significance between groups (*p*<0.05). The comparison of fold expression between mRNAs and their corresponding proteins are presented as the mean ± standard error of the mean (SEM). The correlation coefficient (r^2^) was calculated by Spearman correlation coefficient analysis for establishing the relationship between mRNA and the corresponding protein. The significance of the correlation coefficient was also calculated from an F test.

### The Microarray Data in Public Database

Our whole microarray results were deposited in Gene Expression Omnibus (GEO; http://www.ncbi.nlm.nih.gov/projects/geo/). GSE17400 is the assigned accession number.
